# Feasibility and accuracy of relative electron density determined by virtual monochromatic CT value subtraction at two different energies using the gemstone spectral imaging

**DOI:** 10.1186/1748-717X-8-83

**Published:** 2013-04-09

**Authors:** Toshiyuki Ogata, Takashi Ueguchi, Masashi Yagi, Sachiko Yamada, Chikako Tanaka, Ryota Ogihara, Fumiaki Isohashi, Yasuo Yoshioka, Noriyuki Tomiyama, Kazuhiko Ogawa, Masahiko Koizumi

**Affiliations:** 1 Department of Radiology, Osaka University Hospital, 2-15 Yamadaoka, Suita, Osaka, Japan; 2 Department of Radiation Oncology, Osaka University Graduate School of Medicine, 2-2 Yamadaoka, Suita, Osaka, Japan; 3 Department of Radiology, Osaka University Graduate School of Medicine, 2-2 Yamadaoka, Suita, Osaka, Japan; 4 Division of Medical Physics, Oncology Center, Osaka University Hospital, 2-15 Yamadaoka, Suita, Osaka, Japan

**Keywords:** Gemstone spectral imaging, Monochromatic images, Relative electron density, Dual energy, Computed tomography

## Abstract

**Background:**

Recent work by Saito (2012) has demonstrated a simple conversion from energy-subtracted computed tomography (CT) values (ΔHU) obtained using dual-energy CT to relative electron density (RED) via a single linear relationship. The purpose of this study was to investigate the feasibility of this method to obtain RED from virtual monochromatic CT images obtained by the gemstone spectral imaging (GSI) mode with fast-kVp switching.

**Methods:**

A tissue characterization phantom with 13 inserts made of different materials was scanned using the GSI mode on a Discovery CT750 HD. Four sets of virtual monochromatic CT images (60, 77, 100 and 140 keV) were obtained from a single GSI acquisition. When we define Δ HU in terms of the weighting factor for the subtraction α, Δ HU ≡ (1 + α)H - αL (H and L represent the CT values for high and low energy respectively), the relationship between Δ HU and RED is approximated as a linear function, *a* × Δ HU/1000 + b (a, b = unity). We evaluated the agreement between the determined and nominal RED. We also have investigated reproducibility over short and long time periods.

**Results:**

For the 13 insert materials, the RED determined by monochromatic CT images agreed with the nominal values within 1.1% and the coefficient of determination for this calculation formula was greater than 0.999. The observed reproducibility (1 standard deviation) of calculation error was within 0.5% for all materials.

**Conclusions:**

These findings indicate that virtual monochromatic CT scans at two different energies using GSI mode can provide an accurate method for estimating RED.

## Introduction

Computed tomography (CT) images are used as fundamental input data for most modern radiotherapy treatment planning systems. CT data not only provide anatomic information to delineate target volumes and organs at risk, but also apply corrections to dose calculation to account for tissue inhomogeneities during the radiation treatment planning procedure. These corrections are based on the determination of a relationship between the tissue electron density and its corresponding Hounsfield units (HU) [1].

Dual energy CT (DECT) is one of the most promising imaging techniques with potential clinical applications [2]. DECT has two major advantages compared with conventional single-source CT systems. First, this modality makes it possible to obtain virtual monochromatic images at an arbitrary energy and improved material decomposition such as the separation of iodine from the image [3]. Second, this modality can reduce beam hardening artifacts [4]. A CT value obtained by conventional CT using polychromatic x-rays could have greater uncertainty because of this beam hardening effect [5].

Saito demonstrated a simple conversion from the energy-subtracted CT values (ΔHU) obtained by DECT to the relative electron density (RED) via a single linear relationship [6]. His method is quite simple and accurate, but it requires polychromatic images at different tube potentials. This could limit its application for some dual-energy strategies in which polychromatic dual-energy images are not available. The purpose of this study is to investigate the feasibility of this method to obtain RED from virtual monochromatic images obtained by the gemstone spectral imaging (GSI) mode with rapid kVp-switching single-source DECT. Rapid kVp-switching single-source DECT is capable of alternating hundreds of times per second between low and high (80 and 140 kVp, respectively) tube voltage. This DECT has a new garnet crystal scintillator detector with a much faster optical response compared to typical gadolinium-oxysulfide CT detectors [7]. Since reproducibility of the determined RED is an indicator of the CT value integrity and a prerequisite for radiation therapy treatment, we also investigated the reproducibility of this method over short and long time periods.

## Materials and methods

### Dual energy CT acquisition

In this study, a rapid kVp-switching single-source DECT (Discovery CT750 HD scanner, GE Healthcare, Milwaukee, WI) was used to obtain virtual monochromatic images. A tissue characterization phantom Gammex 467 (Gammex Inc., Middleton, WI) with 13 inserts made of different materials was scanned. The CT scan was performed with the following parameters: 1.0-second tube rotation, 600 mAs tube current, 2.5 mm slice thickness, and 50 cm field of view. We used four sets of monochromatic images at 60 (relatively low), 77, 100 and 140 keV (highest). The 77 keV was close to the effective energy of a 120 kVp polychromatic x-ray according to the specification. The monochromatic and 120 kVp polychromatic images were reconstructed using GSI and Regular mode, respectively. The method by which the GE scanner synthesizes monochromatic CT values from material density images has been described in detail elsewhere [8]. Monochromatic CT image is obtained from the mass attenuation coefficients and density images of the two basis materials with a normalization process by water attenuation coefficient for the desired energy. This study was conducted under the regulations of the Institutional Review Board of our institution.

### Simulation

The dual-energy subtraction for converting CT numbers to RED method developed by Saito [6] was used in this study. According to this method, RED can be calculated simply using CT images at two different energies as follows:

a×ΔHU/1000+b,

where ΔHU is a dual-energy subtracted quantity defined as (1 + α)H - αL (where α is the weighing factor for the subtraction, and H and L represent the CT values for high and low energy respectively), and a and b are unity. The mean CT numbers in HU were measured for each region of interest using ImageJ software (National Institute of Health, Maryland).

Short-term and long-term reproducibility were evaluated by repeating the same measurement every two hours between 9 AM to 5 PM and performing five additional scans at 1-week intervals. The standard deviation of the error in the obtained relative electron density in five successive measurements was calculated and used to evaluate the reproducibility.

### Statistical methods

The difference in RED between nominal and calculated value by DECT was calculated using the following formula: (calculated value-nominal value)/nominal value × 100. For short-term and long-term reproducibility evaluation, each value is presented as a mean error in RED ± standard deviation.

## Results

**Figure 1:**
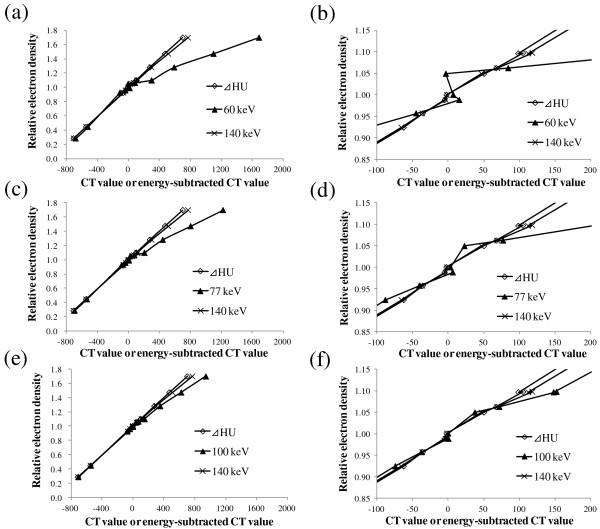
**Relationship between CT value or energy-subtracted CT value of GSI scan images at (a) 60, (c) 77, (e) 100 and 140 keV and RED.** (**b**) (**d**) (**f**) Magnified view of (**a**), (**c**), (**e**), respectively.

Figure 1 shows the relationship between the CT value or energy-subtracted CT value of the GSI scan images and RED. The relationship between the CT and RED became more linear with increasing radiation energy. The relationship between the ΔHU of the GSI scan images and RED was found to be linear with the determination coefficient R^2^ being greater than 0.999. The obtained a and b ranged from 1.004-1.008 and 0.993-0.999, respectively, indicating that these values are close to unity.

**Figure 2:**
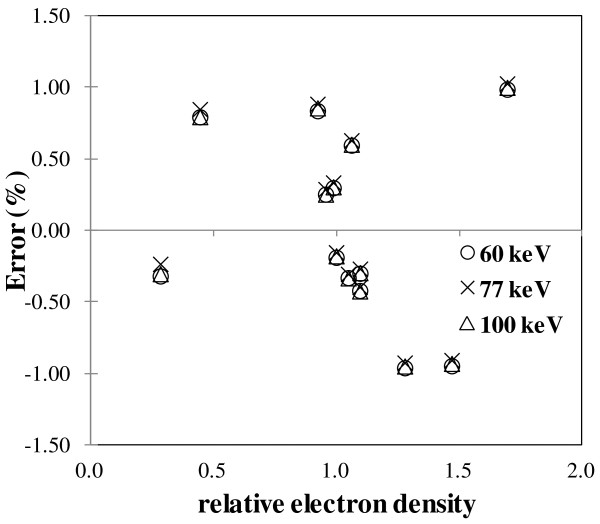
The error in the two different energy virtual monochromatic images subtraction algorithm relative to the nominal value plotted against RED.

The error in the RED determined by the monochromatic CT scan value subtraction at two different energies using GSI relative to the nominal value is shown in Figure 2. The RED obtained from GSI measurements shows ±1.1% agreement with the nominal values for all inserts. The errors in the two different energy monochromatic images subtraction algorithm relative to the nominal value did not show any energy dependence for low kV scans.

**Figure 3:**
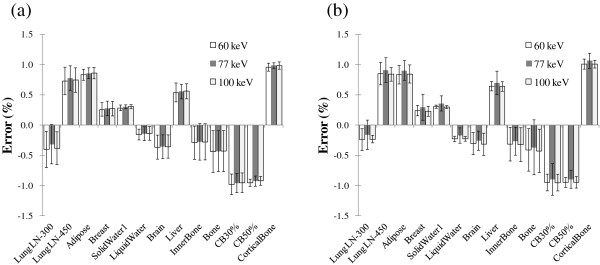
**The error in the two different energy virtual monochromatic images subtraction algorithm relative to the nominal RED value over short and long term-period.** Error bars present one standard deviation obtained from five sets of measurements at (**a**) 2 hours (short-term) and (**b**) 1-week intervals (long-term).

Figure 3 represents the standard deviation for calculation error over short and long time periods. The standard deviation of calculation error over short and long time periods was less than 0.5% for all materials. The reproducibility of the five scans taken at 1-week intervals was comparable to that of the five scans taken at two-hour intervals. The results showed a standard deviation of 0.02 - 0.45; hence, the long-term reproducibility was as good as the short-term reproducibility. The observed calculation error reproducibility was nearly independent of the radiation energy for low kV scans.

## Discussion

Several studies have determined electron density and effective atomic number using the DECT technique [9,10]. However, a practicable dual-energy method that can precisely calculate electron density has not yet been established. We investigated the accuracy of RED obtained by the GSI mode with rapid kVp-switching single-source DECT using the algorithm developed by Saito [6], which is a simple method that converts ΔHU to RED. An advantage of this is that we do not need a priori knowledge regarding the detected x-ray spectra of the CT scanners. We confirmed that the RED obtained from GSI measurements show around ±1.0% agreement with the nominal values for all inserts. IPEM 81 recommends that agreement for electron density should be within 1% for water and within 2% for lung and bone compared with their true values [11]. Agreement for determined RED in our study satisfied the IPEM 81 tolerance levels. These errors are comparable to those of Saito who used a dual-source DECT. The advantages of fast kVp switching CT systems are precise temporal view registration, helical and axial scan, and a 50 cm field of view compared to dual-source DECT [12].

We also investigated the reproducibility of the determined RED over short and long time periods because it is a prerequisite for accurate dose calculations. In the 1980s, the DECT technique had not been used widely in clinical situations due to its lower spatial resolution, unstable CT values, and insufficient tube currents at the low tube voltages of the early CT scanners [13]. We observed that one standard deviation of calculation error was within 0.5% for all materials over the short and long time periods, indicating that no significant variation was observed over the time of the study.

We recognize several limitations in our study. First, we did not evaluate the dependence on scan object size which influences the beam hardening effect. Saito examined the effect of object size on converting ΔHU to RED and confirmed no dependence on the object size [6]. Second, we did not scan high electron density metal, which significantly affects dose distribution in radiation treatment. Further intensive studies are needed to confirm the feasibility of this method for converting ΔHU to RED when using virtual monochromatic images obtained by the GSI mode.

In conclusion, a virtual monochromatic CT scan at two different energies using the GSI mode provides an accurate method for estimating RED.

## Competing interests

The authors declare that they have no competing interests.

## Authors’ contributions

TO performed experiments and drafted the manuscript. TU conceived of the study and participated in its design and coordination and helped to draft the manuscript. MY, SY, RO, and CT collected the data and performed the data analysis. FI, YY, NT, MK, and KO helped to finalize the manuscript. All authors read and approved the final manuscript.
